# Coronary artery bypass grafting in a patient with situs inversus totalis: a case report

**DOI:** 10.1186/s13019-022-01807-9

**Published:** 2022-03-27

**Authors:** Atsushi Oi, Wataru Tatsuishi, Jun Mohara, Toshikuni Yamamoto, Tomonobu Abe

**Affiliations:** Division of Cardiovascular Surgery, Department of General Surgical Science, Gunma University, 3-39-15 Showa, Maebashi, Gunma 371-8511 Japan

**Keywords:** Situs inversus totalis, Dextrocardia, Coronary artery bypass grafting

## Abstract

**Background:**

Coronary artery bypass grafting in situs inversus totalis patients has been seldom reported in the literature.

**Case presentation:**

A 76-year-old woman visited our hospital for chest pain and dyspnea that had started about 5 years earlier. Coronary angiography revealed triple-vessel disease, and computed tomography showed situs inversus totalis. Coronary artery bypass grafting was performed. In this case, the main operating surgeon stood on the right side of the patient until cardiopulmonary bypass was established and then switched positions to the left side of the patient for anastomosis.

**Conclusion:**

CABG was successfully completed in a patient with situs inversus totalis. The position shift helped improve the safety and ease of the surgery.

## Background

Dextrocardia with situs inversus totalis is a rare congenital anomaly in which all organs are mirrored compared to their normal localization [1]. We herein report a patient with situs inversus totalis who underwent coronary artery bypass grafting (CABG). We believe that the position of the operating surgeon is important in this clinical setting.

## Case presentation

A 76-year-old woman visited our hospital for chest pain and dyspnea. The symptoms had started about five years before the visit and had gradually worsened. Her medical history included diabetes mellitus and hyperlipidemia. At the time of admission, her symptoms were Canadian Class 3. Her heart rate was 73/min, and her blood pressure was 150/45 mmHg. No rales or murmur were heard on auscultation.

**Fig. 1:**
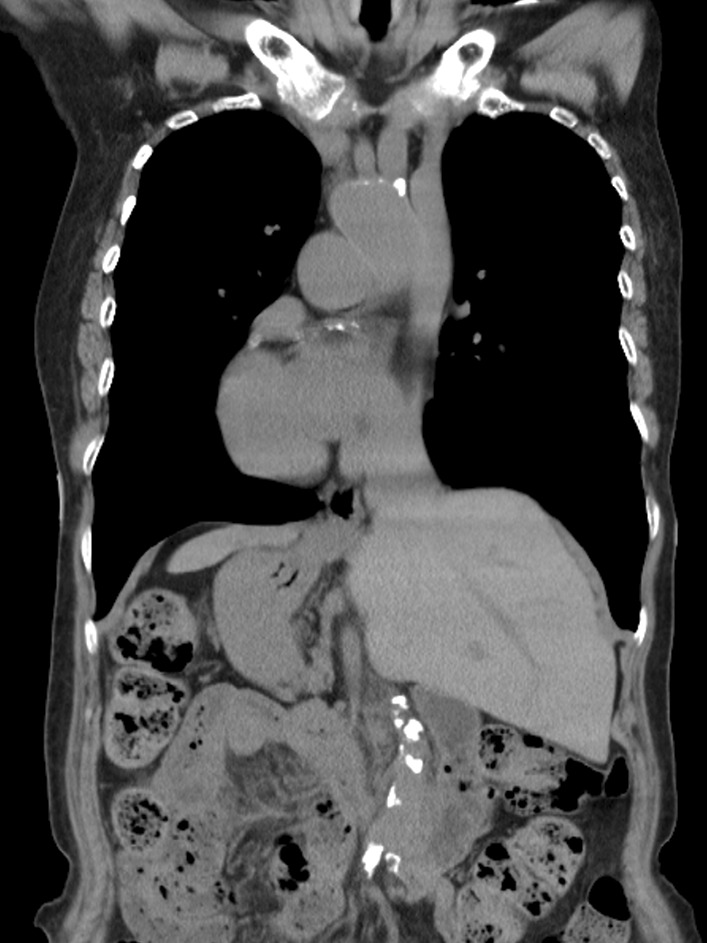
Preoperative computed tomography. All organs, including the cardiovascular system, are mirrored compared to their normal localization

**Fig. 2:**
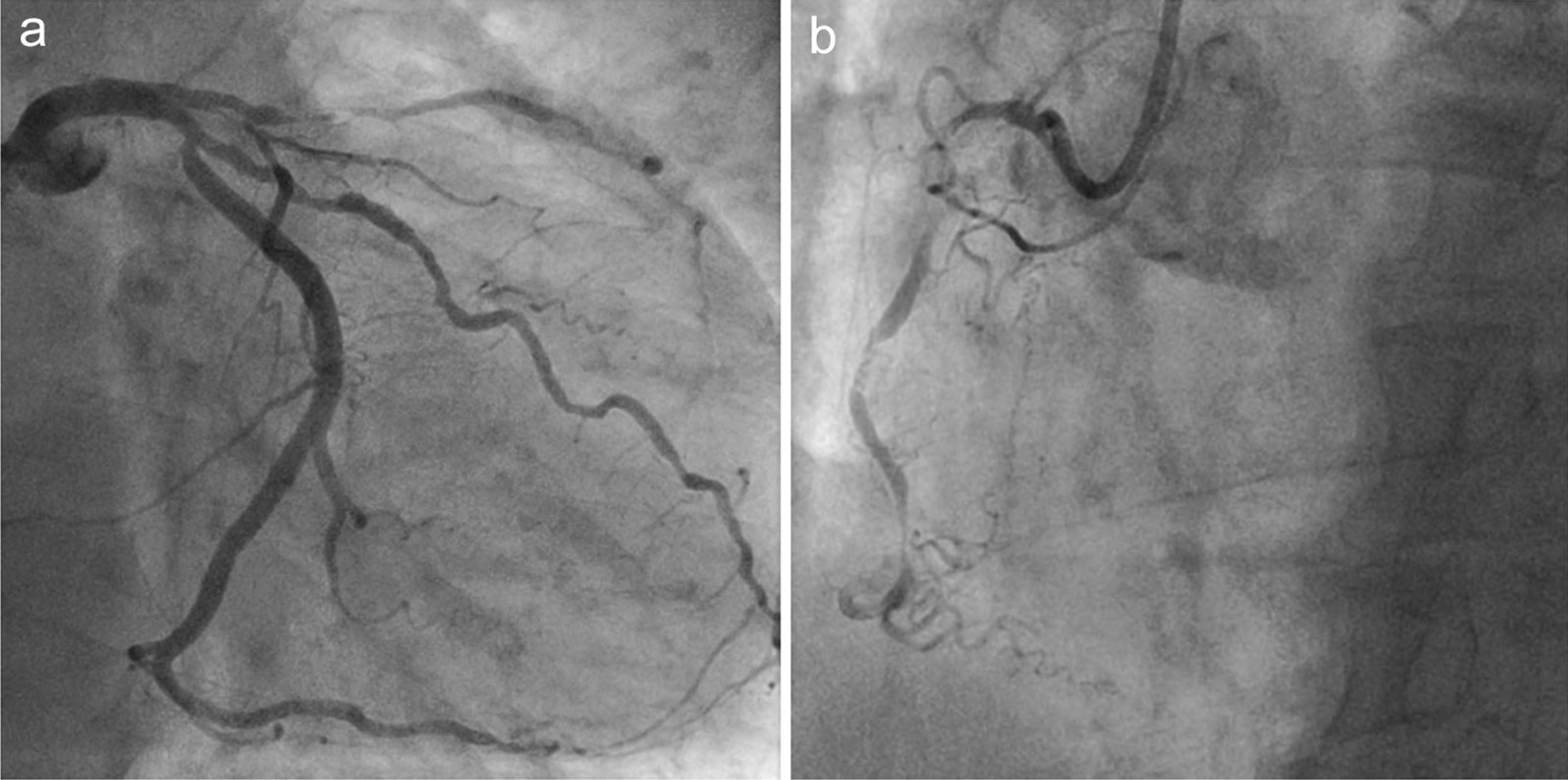
Preoperative coronary angiography. **a** Stenosis of the left anterior descending artery by 99%, stenosis of the left circumflex artery by 90%. **b** Stenosis of the right coronary artery by 99%

Electrocardiography with right chest lead showed ST depression in the V1r to V4r lead and ST elevation in the aVL lead. Transthoracic echocardiography revealed diffuse hypokinesis with a left ventricular ejection fraction of the 35%. Blood test findings were within normal limits. Computed tomography (CT) confirmed highly calcific coronary arteries and situs inversus totalis (Fig. 1). Coronary angiography demonstrated 99% stenosis of the proximal part of the morphologic left anterior descending artery (LAD), 99% stenosis of the right coronary artery (RCA), and 90% stenosis of the left circumflex artery (LCx) (Fig. 2). The treatment choice was discussed among the heart team, and CABG was recommended to the patient.

**Fig. 3:**
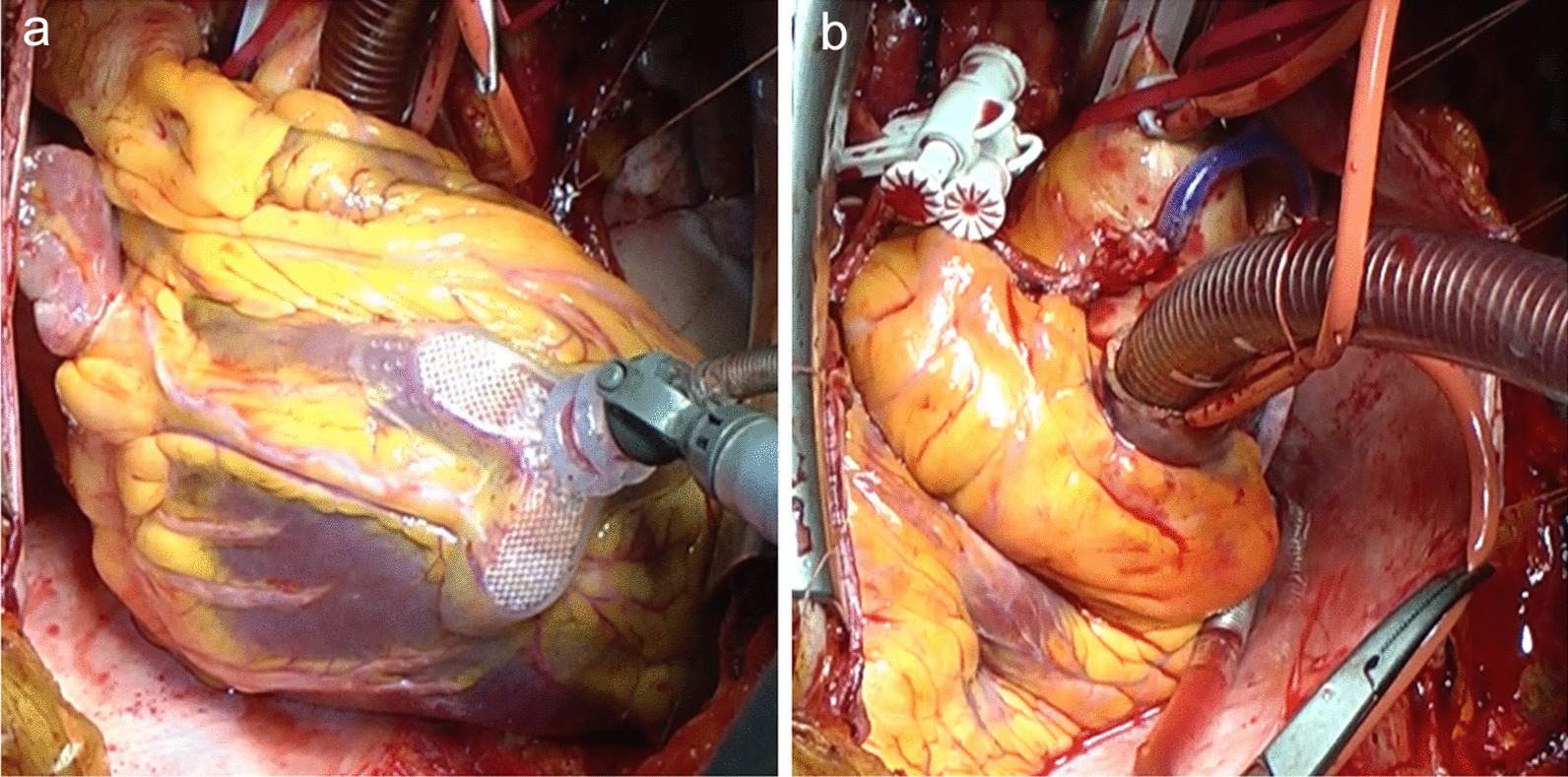
Intraoperative photographs. **a** View of the LCx. Approaching the anastomosis site from the right side of the patient is difficult. **b** End of the anastomosis

During the operation, the surgeon first stood on the right side of the patient. After median sternotomy was performed, the left and right internal thoracic arteries (LITA and RITA) and saphenous vein graft (SVG) were harvested. Cardiopulmonary bypass was established by cannulation of the aorta and the physiological right atrium. At this time, the surgeon switched to the left side of the patient, placed a root cannula, and then cross-clamped the aorta. The SVG was anastomosed to the RCA, and the LITA was anastomosed to the LCx as free grafts. Finally, the RITA was anastomosed in situ to the LAD (Fig. 3). Surgery was completed without any problems. She was extubated four hours after surgery.

The post-operative course was uncomplicated. Post-operative coronary artery angiography showed a sufficient flow.

## Discussion

Dextrocardia in combination with situs inversus totalis is a rare congenital anomaly, with a frequency of 1:10,000 [1, 2]. A total of 20% of situs inversus totalis patients are associated with Kartagener’s syndrome. Whereas cardiac abnormalities associated with isolated dextrocardia occur frequently, dextrocardia with situs inversus is associated with < 10% of cardiac abnormalities and has shown equal frequency to the normal population in terms of coronary artery disease [3]. Fabricius et al. first reported a case of dextrocardia in 1606, and Irvin et al. performed CABG for dextrocardia for the first time in 1980 [4, 5]. The first case of off-pump CABG for dextrocardia with situs inversus was reported by Tabry et al. in 2001 [6].

The primary point of argument concerning CABG for dextrocardia involves the standing position of the operating surgeon and the grafting design. In our search of MEDLINE using the PubMed interface, 26 of 37 cases in which CABG was used for dextrocardia between 1981 and 2021 referred to the standing position, with surgeons standing on the left side in 16 cases, the right side in 7 cases, and both sides in 3 cases [Table 1]. It is important for surgeons to be able to perform their operations easily, so the surgeon in the present case stood on the usual right side until cardiopulmonary bypass was established and then moved to the left side for anastomosis. This approach was particularly effective for anastomosing the free LITA to the LCx, as it is very difficult to perform such anastomosis from the same side of the left ventricular apex. The RITA was anastomosed to the LAD, which was more frequently used in previous case reports of CABG for dextrocardia. RITA-to-LAD anastomosis should be the first choice, as in cases of dextrocardia, this is considered theoretically equal to LITA-to-LAD anastomosis, which has been confirmed to have long-term patency [7]. Off-pump coronary bypass appears to be a simple and feasible option for dextrocardia when the surgeon has sufficient experience. We usually use a pump for uncomplicated multivessel bypass procedures in our institution and it was used in the present case because we wanted to avoid the risk of sudden hemodynamic compromise during off-pump bypass in this case with an anatomical abnormality.

**Table 1:** Case reports referring to CABG in patients with situs inversus

Case	Author	Year	Operation	Pump	Surgeon’s position	Conduits
1	Grey	1981	CABG × 5	On	Not mentioned	SVG
2			CABG × 2	On	Not mentioned	SVG
3			CABG × 2	On	Not mentioned	SVG
4	Irvin	1982	CABG × 3	On	Not mentioned	SVG
5	Moreno-Cabral	1984	CABG × 3	On	Not mentioned	SVG
6	Abensur	1988	CABG × 1	On	Not mentioned	RITA
7	Mesa	1995	CABG × 1	On	Not mentioned	RITA
8	Wong and Chong	1999	CABG × 3	On	Left	RITA, SVG
9	Totaro	2001	CABG × 3	On	Not mentioned	RITA, SVG
10	Tabry	2001	CABG × 4	Off	Left	Both ITAs, SVG
11	Naik	2002	CABG × 2	On	Left	RITA, SVG
12	Erdil	2002	CABG × 2	On	Left	RITA, SVG
13	Stamou	2003	CABG × 2	Off	Both sides	RITA, SVG
14	Bonde	2003	CABG × 2	Converted	Left	RITA, SVG
15	Chui	2003	CABG × 2	On	Left	RITA, Radial artery (RA)
16	Bonanomi	2004	CABG × 2	Off	Not mentioned	RITA, SVG
17	Abdullah	2004	CABG × 3	Off	Right	SVG
18	Kuwata	2004	CABG × 5	Off	Left	Both ITAs, Both RAs
19	Cobiella	2005	CABG × 2, AVR	On	Right	RITA, SVG
20	Baltalarli	2006	CABG × 3	On	Not mentioned	LITA, SVG
21	Poncelet	2006	CABG × 3	On	Both sides	Both ITAs, Gastro-epiploic artery (GEA)
22	Ennker	2006	CABG × 2	Off	Left	RITA
23	Karimi	2007	CABG × 3	On	Right	RITA, SVG
24			CABG × 4	On	Right	RITA, SVG
25	Pego-Fernandes	2007	CABG × 5	On	Left	RITA, SVG
26	Saadi	2007	CABG × 3	On	Left	RITA, SVG
27	Chakravarthy	2008	CABG × 2	Off	Right	LITA, RA, SVG
28			CABG × 3	Off	Both sides	RITA, SVG
29	Yamashiro	2009	CABG × 3	Off	Right	Both ITAs, RA
30	Kuthe	2011	CABG × 3, VSD closure	On	Right	SVG
31	Dabbagh	2011	CABG × 3	Off	Left	RITA, SVG
32	Yuan	2015	CABG × 2	Off	Left	RITA, SVG
33			CABG × 3	Off	Left	RITA, SVG
34	Kono	2016	CABG × 1, AVR	On	Left	SVG
35	Subash	2017	CABG	Not mentioned	Left	RITA, SVG
36	Zhigalov	2019	CABG × 2	On	Left	Both ITAs
37	Cheng	2021	CABG × 4	On	Not mentioned	LITA, SVG

Twenty-six of 37 cases of CABG for dextrocardia between 1981 and 2021 referred to the standing position, with surgeons standing on the left side in 16 cases, the right side in 7 cases, and both sides in 3 cases. All cases mention bypass grafts. SVGs were used in 29 cases, RITAs were used in 26 cases, LITAs were used in 8 cases, and RAs were used in 4 cases; GEA was only used in one case

## Conclusions

CABG was successfully completed in a patient with situs inversus totalis. The operation was performed safely by switching the surgeon’s standing position and then selecting the most appropriate bypass grafts.

## Data Availability

Not applicable.

## Abbreviations

CABG: Coronary artery bypass grafting
CT: Computed tomography
LAD: Left anterior descending artery
RCA: Right coronary artery
LCx: Left circumflex artery
LITA: Left internal thoracic artery
RITA: Right internal thoracic artery
SVG: Saphenous vein graft
RA: Radial artery
GEA: Gastroepiploic artery
